# Adapting diabetes medication for low carbohydrate management of type 2 diabetes: a practical guide

**DOI:** 10.3399/bjgp19X704525

**Published:** 2019-07

**Authors:** Campbell Murdoch, David Unwin, David Cavan, Mark Cucuzzella, Mahendra Patel

**Affiliations:** Wincanton Health Centre, Wincanton; chief medical officer, Digital Diabetes Media, Coventry, UK.; Norwood Surgery, Southport, UK.; London Medical, London, UK.; West Virginia University School of Medicine, WVU Center for Diabetes and Metabolic Health, Morgantown, US.; Medical School, University of Sheffield, Sheffield, UK.

## INTRODUCTION

The pathological changes associated with type 2 diabetes (T2D) can be reversed through lifestyle measures, in some cases leading to remission.^1^ The low carbohydrate diet (LCD) is recognised as an effective option that is clinically inexpensive with few side effects.^2^ Many patients are achieving significant improvements in glycaemic control, with associated reduction in drug costs from cessation of hypoglycaemic agents.^3^ Digital-technology behaviour change solutions for T2D remission are being delivered at scale.^4^ Primary care clinicians need to be competent to adjust diabetes medications appropriately in individuals who follow an LCD.

## THE LCD IN TYPE 2 DIABETES

An LCD comprises <130 g of digestible carbohydrates per day.^5^ Digestible carbohydrate refers to sugars and complex carbohydrates such as starch, which is digested to glucose. Aligned with national guidance, carbohydrate choices in an LCD will typically be higher fibre and low glycaemic index (GI).^6^ Reduced total carbohydrate ingestion and low GI choices give the LCD a low glycaemic load (GL). In T2D the GI and GL of food consumed is a determinant of blood glucose level and thus the requirement for hypoglycaemic medication.

## DIABETES MEDICATIONS AND AN LCD

Blood glucose levels typically fall substantially when an individual adopts an LCD. This article discusses key considerations regarding hypoglycaemic medications for an LCD and provides practical suggestions to prescribers. The recommendations are developed from the experience of the authors, discussion with experts, and the pharmacodynamics of the medications. Antihypertensive medications are not discussed in this article but clinicians need to be aware that an LCD can improve blood pressure, and antihypertensives may need to be adjusted.

When deciding the safety and appropriateness of T2D medications with an LCD there are three key clinical considerations: Is there a risk of the drug causing hypoglycaemia or other adverse event?; What is the degree of carbohydrate restriction?; Once carbohydrate is reduced does the drug continue to provide health benefit, and if so are the potential drug benefits greater than or less than possible risks and side effects?

## MEDICATIONS THAT CREATE A RISK OF HYPOGLYCAEMIA

### Sulphonylureas and meglitinides

Sulphonylureas (for example, gliclazide) and meglitinides (for example, repaglinide) should be reduced or stopped when an LCD is commenced. An initial dosage reduction of at least 50% is typically appropriate, with further reductions according to blood glucose response. There may be a period of short-term hyperglycaemia while the individual adapts to an LCD.

### Insulins

Practical expertise suggests a 50% reduction of daily insulin dose at initiation of the LCD is appropriate in most cases. In individuals whose HbA1c is markedly elevated, a smaller reduction of perhaps 30% may be appropriate, with further reductions over time. For individuals on a basal-bolus regimen it is preferential to reduce or stop bolus insulin. In individuals on a mixed insulin or basal insulin alone each dose can be reduced by 30–50% at the start of LCD. Some patients can expect to come off insulin completely, over days or months, as insulin resistance resolves. Improving blood glucometer readings can guide the down-titration of insulin.

It should be cautioned that some patients may have an insulin insufficiency form of diabetes, such as latent autoimmune diabetes of adults. Although the LCD enables a reduction in insulin dosage it should not be completely stopped in this cohort of patients. Endogenous insulin insufficiency is more likely in patients who were not overweight at the time of diagnosis of diabetes, and it may be present in some with longstanding T2D. Over-reduction in insulin dosage in these patients would lead to significant hyperglycaemia, and thus further dosage reduction would be avoided. It is recommended that additional investigation and expert advice is sought in cases of doubt as per good medical practice.

## MEDICATIONS THAT RISK KETOACIDOSIS

### SGLT2 inhibitors (‘flozins’)

These carry a risk of causing ketoacidosis if a patient has significant insulin insufficiency, with any diet. SGLT2i-induced ketoacidosis may occur with a normal blood glucose, which heightens the risk of the life-threatening condition going unrecognised. Consider the possibility of insulin insufficiency, including in those who could have been misdiagnosed with T2D. In a community setting, for safety and simplicity, it may be appropriate for most patients to stop their SGLT2i when an LCD is initiated. This removes the SGLT2i-induced ketoacidosis risk, and additionally the effectiveness of the LCD means the benefit of a SGLT2i is diminished. (Note: a very LCD, typically <30–50 g carbohydrate/day, can produce a physiologically normal state of ketosis, which should not be confused with the pathological state of ketoacidosis.)

## MEDICATIONS THAT POSE NO EXCESS RISK WITH AN LCD

### Metformin

This is safe to continue and in some patients metformin continues to offer favourable benefits. Up to 25% of patients experience gastrointestinal side effects from metformin.

### GLP-1 agonists (‘-enatide’, ‘-glutide’)

These are safe to continue, with the beneficial actions of increased satiety and slowed gastric emptying, and possibly cardiovascular benefit. With a sustained LCD patients may be able to stop their GLP-1 agonist.

### Thiazolidinediones (‘glitazones’)

These are safe to continue from a short-term perspective. Concerns exist over their long-term safety including: bladder cancer, heart failure, and bone mineral density. Thiazolidinediones are also known to cause weight gain. It is recommended to stop thiazolidinediones as soon as blood glucose levels allow.

### DPP-4 inhibitors (‘gliptins’)

These are safe to continue. However, clinical experience agrees that these seem to have little blood glucose-lowering effect in the context of an LCD.

### Acarbose

This is safe to continue. But, on commencing an LCD the reduced starch ingestion means the patient can usually stop acarbose.

### Blood glucose testing strips

Structured self-monitoring of blood glucose, such as paired pre- and post-meal testing, can be very helpful by providing rapid feedback on how foods affect blood glucose as a patient adopts an LCD, and to inform whether medication doses can be reduced further. Patients on drugs that risk hypoglycaemia should have access to adequate testing strips.

## CONCLUSION

The LCD is an increasingly popular option for managing T2D that can lead to improvements in the condition, reduced medication burden, and (where needed) weight loss. Primary care clinicians need to be competent in adjusting diabetes medications to achieve safe and effective care (Box 1).

**Box 1.** Summary guidance on adapting diabetes medication for low carbohydrate management of type 2 diabetes

**Drug group**	**Hypo risk?**	**Clinical suggestion**
Sulphonylureas (for example, gliclazide) and meglitinides (for example, repaglinide)	Yes	Reduce/stop (if gradual carbohydrate reduction then wean by halving dose successively)
Insulins	Yes	Reduce/stop. Typically wean by 30–50% successively. Beware insulin insufficiency^a^
SGLT2 inhibitors (flozins)	No	Ketoacidosis risk if insulin insufficiency. Usually stop in community setting
Biguanides (metformin)	No	Optional, consider clinical pros/cons
GLP-1 agonists (-enatide/-glutide)	No	Optional, consider clinical pros/cons
Thiazolidinediones (glitazones)	No	Usually stop, concerns over long-term risks usually outweigh benefit
DPP-4 inhibitors (glipitins)	No	Usually stop, due to lack of benefit
Alpha-glucosidase inhibitors (acarbose)	No	Usually stop, due to no benefit if low starch/sucrose ingestion
Self-monitoring blood glucose	N/A	Ensure adequate testing supplies for patients on drugs that risk hypoglycaemia. Testing can also support behaviour change (for example, paired pre- and post-meal testing)

a Caution should be taken when reducing insulin if there is clinical suspicion of endogenous insulin insufficiency (Patients with LADA misdiagnosed as T2D; a minority of T2 patients have endogenous insulin deficiency). Consider these possibilities if patient was not overweight at diagnosis. Exogenous insulin should not be completely stopped in these cases. Inappropriate over-reduction of exogenous insulin will lead to marked hyperglycaemia. Hypo = hypoglycaemia. LADA = latent autoimmune diabetes in adults. T2D = type 2 diabetes.

## References

[1] McCombie L, Leslie W, Taylor R (2017). Beating type 2 diabetes into remission. BMJ.

[2] Forouhi NG, Misra A, Mohan V (2018). Dietary and nutritional approaches for prevention and management of type 2 diabetes. BMJ.

[3] Unwin D, Haslam D, Livesey G (2016). It is the glycaemic response to, not the carbohydrate content of food that matters in diabetes and obesity: the glycaemic index revisited. Journal of Insulin Resistance.

[4] Saslow LR, Summers C, Aikens JE, Unwin DJ (2018). Outcomes of a digitally delivered low-carbohydrate type 2 diabetes self-management program: 1-year results of a single-arm longitudinal study. JMIR Diabetes.

[5] Feinman RD, Pogozelski WK, Astrup A (2015). Dietary carbohydrate restriction as the first approach in diabetes management: critical review and evidence base. Nutrition.

[6] National Institute for Health and Care Excellence (2015). Type 2 diabetes in adults: management NG28.

